# A Network Pharmacology Approach to Reveal the Underlying Mechanisms of *Paeonia lactiflora* Pall. On the Treatment of Alzheimer's Disease

**DOI:** 10.1155/2019/8706589

**Published:** 2019-11-16

**Authors:** Qiang Zeng, Longfei Li, Yu Jin, Zongzheng Chen, Lihong Duan, Meiqun Cao, Min Ma, Zhengzhi Wu

**Affiliations:** ^1^Integrated Chinese and Western Medicine Postdoctoral Research Station, School of Traditional Chinese Medicine, Jinan University, Guangzhou 510632, China; ^2^The First Affiliated Hospital of Shenzhen University, Shenzhen Second People's Hospital, Shenzhen 518035, China; ^3^Shenzhen Institute of Geriatrics, Shenzhen 518020, China; ^4^Laboratory of Molecular Pharmacology, Department of Pharmacology, School of Pharmacy, Southwest Medical University, Luzhou 646000, China

## Abstract

**Objective:**

To investigate the potential active compounds and underlying mechanisms of *Paeonia lactiflora* Pall. (PLP) on the treatment of Alzheimer's disease (AD) based on network pharmacology.

**Methods:**

The active components of PLP were collected from Traditional Chinese Medicine System Pharmacology (TCMSP) database, and their possible target proteins were predicted using TCMSP, SwissTargetPrediction, and STITCH databases. The putative AD-related target proteins were identified from Therapeutic Target Database (TTD), GeneCards, and MalaCards database. The compound-target-disease network interactions were established to obtain the key targets about PLP acting on AD by network topology analysis. Then, the function annotation and signaling pathways of key targets were performed by GO and KEGG enrichment analysis using DAVID tools. Finally, the binding capacity between active ingredients and key targets was validated by molecular docking using SystemsDock tools.

**Results:**

There were 7 active compounds involving in 151 predicted targets identified in PLP. Besides, a total of 160 AD-related targets were identified. Among these targets, 30 shared targets of PLP and AD were acquired. After topological analysis of the PLP potential target-AD target network, 33 key targets that were highly responsible for the therapeutic effects of PLP on AD were obtained. Further GO and KEGG enrichment analysis showed that these key targets were significantly involved in multiple biological processes and pathways which participated in cell apoptosis and inflammatory response and maintained the function of neurons to accomplish the anti-AD activity. The molecular docking analysis verified that the 7 active compounds had definite affinity with the key targets.

**Conclusions:**

The ameliorative effects of PLP on AD were predicted to be associated with regulating neural cell apoptosis, inflammatory response, and neurotrophy via various pathways such as PI3K-Akt signaling pathway, MAPK signaling pathway, and neurotrophin signaling pathway.

## 1. Introduction

Alzheimer's disease (AD) is one of the commonest neurodegenerative diseases with high incidence and intricate pathogenesis. Unfortunately, there is no efficacious treatment options for AD patients. AD contributes to about two-thirds of dementia cases and affects more than 5 million people in US [1]. The prevalence of AD is everincreasing with years, and AD has been the sixth primary cause of death in the US, although the AD-induced death emerges on average 8.5 years [2]. The clinical features of AD contain memory loss and cognitive impairment at early stage, subsequent topographical difficulties, and alongside loss of confidence, judgement and attention. As the condition progresses, cognitive deficiency becomes deteriorative and widespread so as to interfere with activities of daily living and bring poor quality of life to the patients and their families [3]. However, the current mainstream treatments for AD such as acetylcholinesterase (AChE) inhibitors and *N*-methyl-D-aspartate (NMDA) receptor antagonists show limited efficacy. The major pathogenic events leading to AD are attributed to the accumulation of insoluble amyloid-*β* (A*β*) to form senile plaque and aggregation of microtubule protein tau in neurofibrillary tangles (NFTs) in neurons [4]. Recently, the clinical trials showed that new drugs targeting A*β* or tau failed to improve cognitive ability and clinical outcomes of AD patients, and thus they were discontinued. It suggested that the efficacy of single-target drug was limited and hard to meet clinical needs [5–7]. It is important and necessary to develop novel drugs with multitargets for AD treatment.

Traditional Chinese Medicines (TCMs) has been used for the treatment of dementia for hundreds of years in China. According to the theory of TCM, AD pertains to long-lasting nutrition deficiency in the brain as a result of obstruction of blood flow and disturbance of “Qi” motion in liver [8]. *Paeonia lactiflora* Pall. (PLP) is a well-known and widely used herbal medicine that can nourish blood and regulate the liver. Modern pharmacological studies disclosed that formulae containing PLP had evident activities in reducing tau aggregation and ameliorating cognition deficits [9–11]. The aqueous and ethanol extracts of PLP exhibited strong inhibition on AChE activity as indicated by IC_50_ values at 20 and 8 *μ*g/ml, which was even stronger than S*alvia miltiorrhiza* Bge. and *Polygonum multijiorum* Thunb., implying great potential for AD treatment [12]. Although paeoniflorin was reported to be one of the active ingredients in PLP which could improve dementia through neuroprotection and anti-inflammation, the other active compounds in PLP and possible multitarget mechanisms were seldom reported [13, 14].

Network pharmacology is an emerging approach for drug discovery. Up to date, this approach has been successfully employed to elucidate the multitarget effects of TCM, which is consistent with the holistic perspective of TCM theory. The use of network pharmacology in the research of TCM integrates phytochemistry, pharmacology, and bioinformatics; effectively bridges the gap between western medicine and traditional medicine; and also greatly facilitates mechanistic studies on the synergistic actions of TCM [15]. In the current study, the active compounds and underlying mechanisms of PLP acting on AD were comprehensively investigated using the network pharmacology approach. The compounds in PLP and their putative target proteins were identified from the public databases. The biological processes and underlying pathways associated with PLP acting on AD were obtained by enrichment analysis. The combination between the active ingredients in PLP and the key targets was evaluated by simulative molecular docking as well. The present study elucidated the potential active compounds and underlying mechanisms of PLP acting on AD and provided theoretic evidence for the multi-ingredient and multitarget effects of PLP, suggesting the feasibility of developing PLP or its active compounds as alternative therapy for AD.

## 2. Materials and Methods

### 2.1. Identification of Chemical Ingredients in PLP

The chemical ingredients in PLP were identified from Traditional Chinese Medicine System Pharmacology (TCMSP, http://lsp.nwu.edu.cn/browse.php) database [16]. The database provides comprehensive information about ingredients in herbs including chemical structure, oral bioavailability, Caco-2 intestinal epithelial permeability, half-life, drug likeness, drug targets, and their association with diseases and interaction network, etc. The pharmacokinetic properties including absorption, distribution, metabolism, and excretion (ADME) are important contributors for bioactivities of drug. In this study, three ADME-related parameters including oral bioavailability (OB) ≥30%, half-life (HL) ≥4, and drug likeness (DL) ≥0.18 were employed to identify the potential active ingredients in PLP. As suggested by TCMSP, the compounds with OB ≥30% and HL ≥4 have good absorption and slow metabolism after oral administration. The compounds with DL ≥0.18 were chemically suitable for drug development.

### 2.2. Prediction of Compound-Related Targets

The compound-related targets were predicted depending on chemical similarities and pharmacophore models via TCMSP, SwissTargetPrediction (http://www.swisstargetprediction.ch/), and STITCH (http://stitch.embl.de/) databases. The probability value of each target marked in SwissTargetPrediction database was used to give a rank list for the targets and evaluate the accuracy of the predictions, whose probability value ≥0.5 was collected in our present study [17]. Besides, confidence score marked in STITCH database provided a reference to define a set of high-confidence interactions between compounds and protein modules [18]. The target proteins with confidence score ≥7 were identified as compound-related targets.

### 2.3. Identification of AD-Related Targets

The known AD-related targets were collected from three databases including TTD (Therapeutic Target Database, https://db.idrblab.org/ttd/), GeneCards (https://www.genecards.org/), and MalaCards (https://www.malacards.org/pages/info).

All the targets obtained above were standardized as gene names and UniProt IDs by searching from UniprotKB (https://www.uniprot.org/) database with “Homo sapiens” species [19].

### 2.4. Network Construction and Topological Analysis

The compound-target network of PLP and disease-target network of AD, as well as PLP potential target-AD target interaction network, was constructed by Cytoscape v3.7.0 software which is a useful tool for analysis and visualization of the biological network. The topological analysis was performed by the network analyzer module of Cytoscape software. Three topological parameters including degree centrality (DC), betweenness centrality (BC), and closeness centrality (CC) were used to estimate the central properties of the nodes in the network. In the PLP potential target-AD target interaction network, DC ≥ median DC, BC ≥ median BC, and CC ≥ median CC were employed to screen the key targets of PLP acting on AD.

### 2.5. GO and KEGG Pathway Enrichment Analysis

DAVID (https://david.ncifcrf.gov/) is an online biological knowledgebase and an analytic tool to extract biological information about gene functional classification, functional annotation, and enriched pathways [20]. Gene Ontology (GO) analysis including biological process, cell component, and molecular function, as well as KEGG pathway enrichment analysis, were performed using DAVID database. GO terms with Bonferroni value <0.05 and KEGG pathways with *P* value <0.05 were considered to have significance.

### 2.6. Validation of the Binding Capacity between Active Ingredients and Key Targets by Molecular Docking

Molecular docking is a useful method for drug targets and drug screening research by mimicking the interactions between compounds and proteins to predict their binding capacity and affinity based on their structures. SystemsDock (http://systemsdock.unit.oist.jp/), a web server for assessing protein-ligand binding property, permits high-precision docking simulation and molecular pathway map for comprehensive characterization of ligand selectivity and interpretation of ligand action on a complex molecular network [21]. The crystal structure of the key targets were obtained from PDB (Protein Data Bank) database, and then the molecular docking results between active ingredients in PLP and key targets were analyzed using systemsDock to validate their binding properties. Docking score is a parameter that is the predicted binding affinity to each of target proteins. Docking score at 5.52 is set as the cutoff to classify the good binding capacity between a compound and protein [21].

## 3. Results

### 3.1. Compound-Target Network of PLP

Total 85 compounds were identified in PLP including terpenoids, glycosides, flavonoids, oleum volatile, and phenols. According to the characteristics of oral bioavailability, half-life, and drug likeness of the compounds, 7 compounds were screened out as the potential active ingredients including *β*-sitosterol, kaempferol, lactiflorin, mairin, paeoniflorigenone, paeoniflorin, and palbinone, which are listed in Table 1. The characteristics of the 85 compounds in PLP are shown in Stable 1.

**Figure 1:**
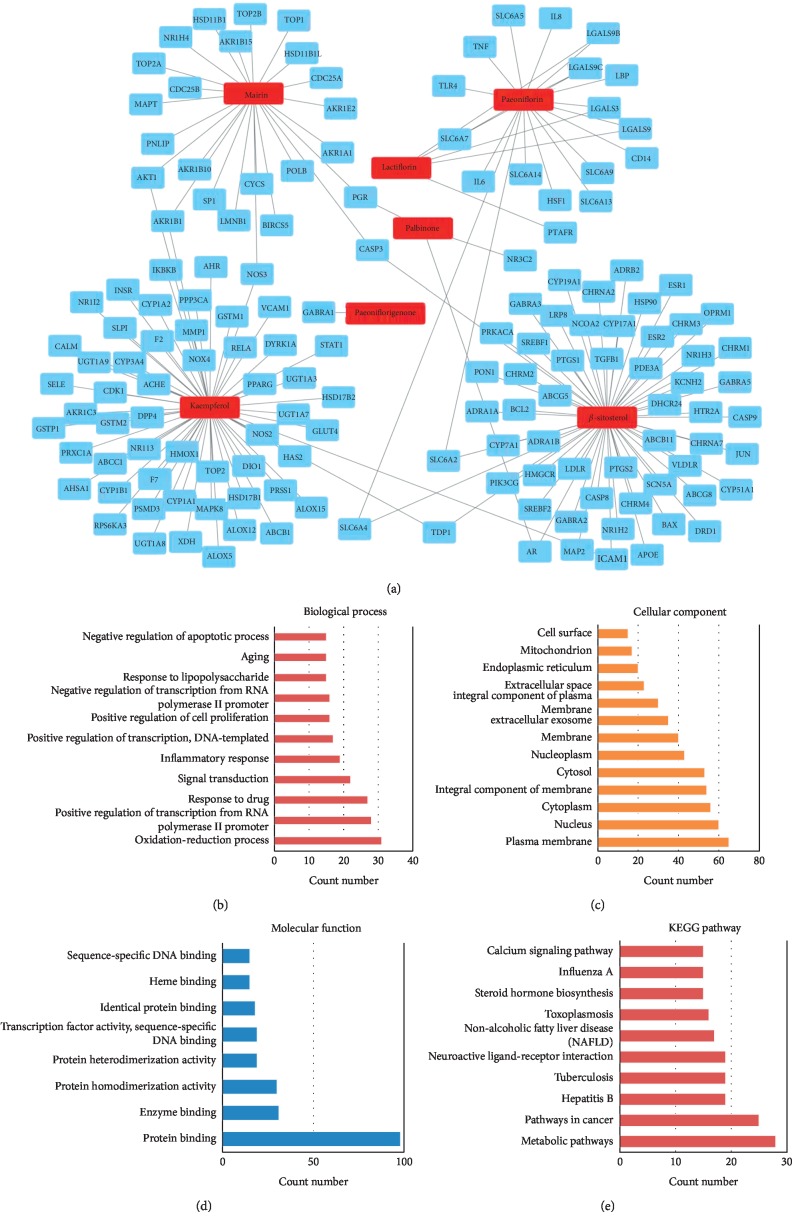
(a) Compound-target network of PLP. Red rectangle nodes represent potential active compounds in PLP, while blue rectangle nodes represent potential targets of PLP. (b)–(d) GO enrichment analysis for potential targets of PLP (count number ≥15). (e) KEGG enrichment analysis for potential targets of PLP (count number ≥15).

From the TCMSP, SwissTargetPrediction, and STITCH databases, a total of 151 compound-related targets were identified. Among these targets, there were 57 targets involved in *β*-sitosterol, 58 targets involved in kaempferol, 5 targets related to lactiflorin, 24 targets related to mairin, 18 targets related to paeoniflorin, 3 targets involved in palbinone, and only one target connected to paeoniflorigenone. These seven compounds also had some shared bioactive targets and connection networks. The targets of each compound are listed in Stable 2, and the compound-target network of PLP is shown in Figure 1(a).

To clarify the characteristics of compound-related targets on molecular function and pathway level, GO function and KEGG pathway enrichment analysis were performed (Figures 1(b)–1(e)). Most of these potential targets existed on the plasma membrane with molecular function of protein binding. Specifically, these targets were involved in biological processes such as oxidation-reduction process, regulation of transcription, response to drug, signal transduction, and inflammatory response. Furthermore, the results of KEGG enrichment analysis demonstrated that there were totally 101 pathways (*P* value <0.05) affected by the active ingredients of PLP. The top 10 (count number ≥15) enriched pathways contained metabolic pathways, pathways in cancer, hepatitis B, tuberculosis, neuroactive ligand-receptor interaction, nonalcoholic fatty liver disease, toxoplasmosis, steroid hormone biosynthesis, influenza A, and calcium signaling pathway.

### 3.2. Disease-Target Network of AD

**Figure 2:**
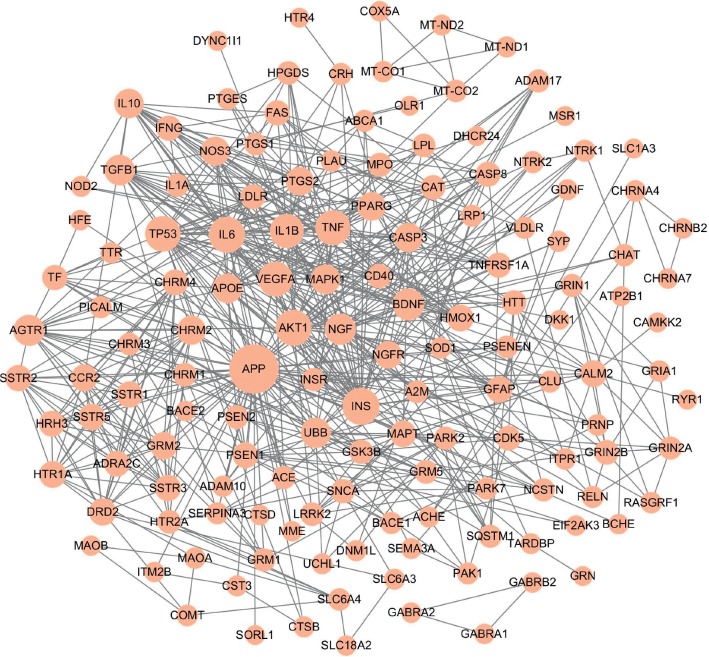
Disease-target network of AD. Pink ellipse nodes represent AD-related targets, and the size of nodes is proportional to degree centrality by topology analysis.

From the TTD, GeneCards, and MalaCards databases, a total of 160 AD-related targets were identified (Stable 3), among which 142 targets showed high interactions (confidence score ≥7) in the protein-protein interactions map generated by the STRING database. The disease-target network of AD was constructed and consisted of 142 nodes (targets with high interactions) and 626 link edges. The central attributes of each node were evaluated by topology analysis. In Figure 2, the size of nodes was proportional to degree centrality. Notably, amyloid precursor protein (APP, degree = 56) which played an essential role in the pathogenesis of AD brain was identified as the most important protein in this disease-target network. In addition, according to both degree and betweenness centrality, INS, AKT1, IL6, TP53, BDNF, and NGF were also recognized as important AD targets.

### 3.3. PLP Potential Target-AD Target Network

**Figure 3:**
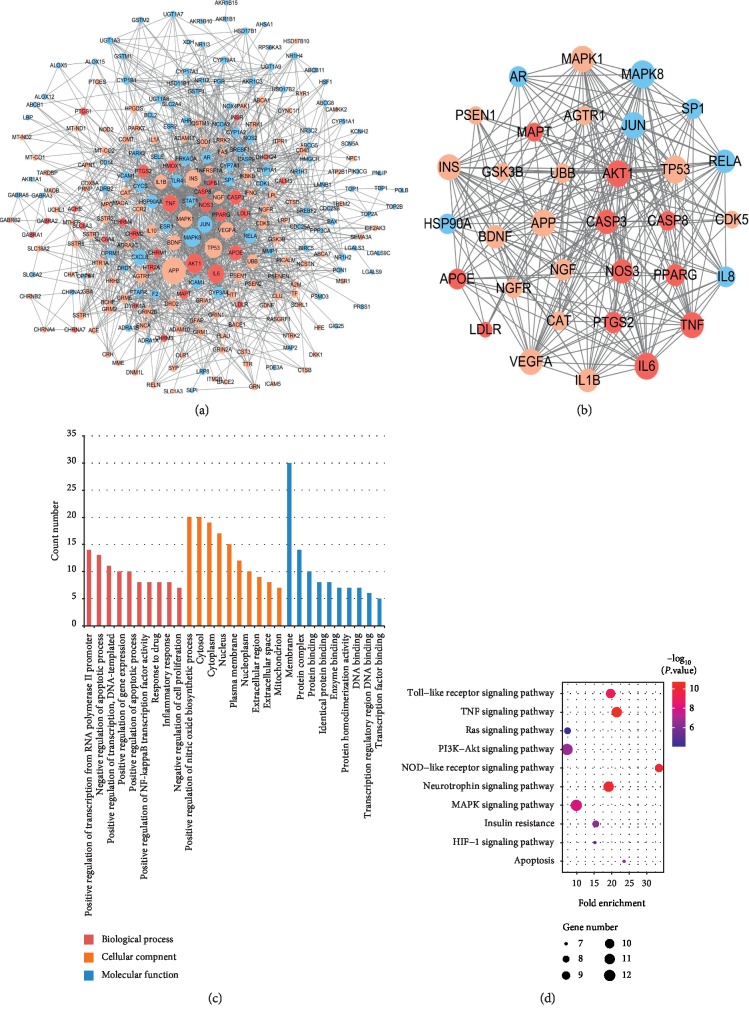
(a) PLP potential target-AD target network. (b) Network of the 33 key targets. Blue ellipse nodes stand for PLP potential targets, pink ellipse nodes represent AD-related targets, and red ellipse nodes represent the shared targets of PLP and AD. The size of nodes is proportional to degree centrality by topology analysis. (c) GO enrichment analysis for 33 key targets. (d) KEGG enrichment analysis for 33 key targets.

To identify the relationship between the PLP potential targets and AD-related targets, their targets were analyzed, and thus 30 shared targets of PLP and AD were acquired. The interaction network of PLP potential target-AD target was established and shown in Figure 3(a). There were 260 targets and 1634 link edges which had high interactions with confidence score ≥7 in the STRING database. Based on criteria of DC ≥ median DC, BC ≥ median BC, and CC ≥ median CC, 33 key targets of PLP acting on AD were obtained (Table 2) and further used to construct the interaction network consisting of 33 nodes and 254 link edges (Figure 3(b)).

These key targets were primarily distributed in cellular components such as cytosol and cytoplasm and mainly involved in biological processes such as regulation of transcription, regulation of apoptotic process, regulation of gene expression, inflammatory response, and regulation of nitric oxide biosynthetic process via molecular function of protein binding by GO enrichment analysis (Figure 3(c)).

The KEGG enrichment analysis was carried out to further explore the underlying mechanisms of PLP on AD. The representative top 10 pathways based on the number of enriched genes as well as fold changes and *P* value are shown in Figure 3(d). These key targets were closely related to PI3K-Akt signaling pathway, MAPK signaling pathway, neurotrophin signaling pathway, TNF signaling pathway, toll-like receptor signaling pathway, etc., which participated in cell apoptosis and inflammatory response and maintained the function of neurons to accomplish the anti-AD activity of PLP.

### 3.4. The Binding Capacity between Active Compounds and Key Targets by Molecular Docking

**Figure 4:**
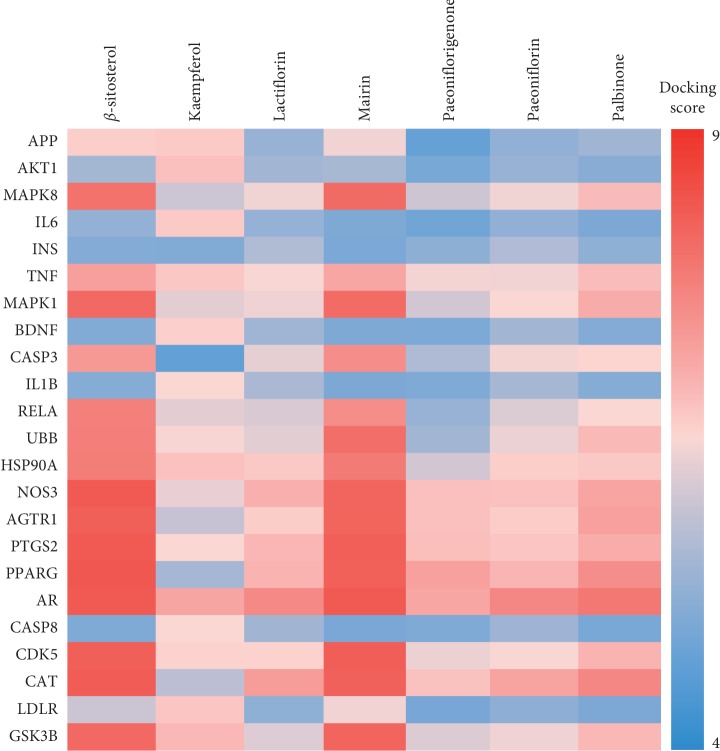
Heat map of binding capacity between the active compounds and key targets by molecular docking. The heat map was depicted based on docking scores.

To further verify the binding capacity between active compounds and key targets, molecular docking through systemsDock was performed. The docking results are shown in Figure 4 based on the docking score which was a negative logarithm of the experimental dissociation/inhibition constant, ranging from 0 to 10 that represented weak to strong binding. The docking scores showed that the active compounds of PLP, especially *β*-sitosterol and mairin, had good binding activity to AD putative targets included in the key targets, indicating the specific action proteins of PLP for AD treatment.

## 4. Discussion

**Figure 5:**
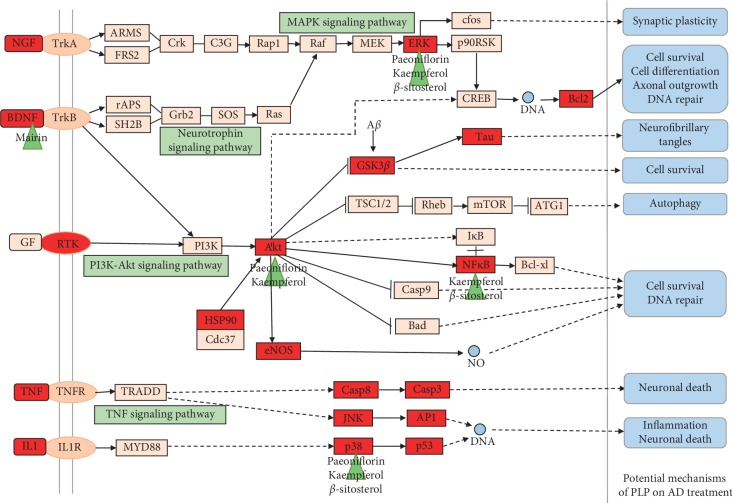
Representative compounds and potential mechanisms of PLP on AD treatment. The green triangle stands for representative compounds in PLP, and the red rectangle stands for the compound-related targets.

According to the theory of TCM, long-term nutrition deficiency in the brain as a result of blood stasis and “Qi” stagnation in liver is one of the pathogenic reasons for AD. PLP is a well-known herbal medicine with a function of nourishing and regulating blood and is commonly used to improve nutrition deficiency in the brain. Among the large numbers of formulae for treatment of dementia, PLP is one of the most widely used herbs and usually works as a monarch herb in a prescription [22]. The present study aimed to explore the potential active compounds and underlying mechanisms of PLP acting on AD and provided theoretic evidence for developing PLP or its active compounds as alternative therapy for AD. The representative compounds and potential mechanisms of PLP on AD treatment are depicted in Figure 5. In the current study, there were 7 compounds including *β*-sitosterol, kaempferol, lactiflorin, mairin, paeoniflorigenone, paeoniflorin, and palbinone identified as the potential active ingredients of PLP, of which the biological activities against AD were reported previously. For example, kaempferol, that is, a natural acetylcholinesterase inhibitor could delay the loss of climbing ability, ameliorate memory deficiency, and reduce oxidative stress and neuroinflammation both in the ovariectomized rat model and transgenic Drosophila model of AD [23–25]. Mairin, also named as betulinic acid, that is, a pentacyclic triterpenoid, could prevent A*β*/streptozotocin-induced spatial and passive avoidance memory deficits and reduce A*β* fibril plaques in the hippocampus region of the AD rat model through protecting microcirculation, alleviating inflammation, and upregulating BDNF expression [26, 27]. Paeoniflorin is reported to improve memory deficits, attenuate amyloidogenesis, prevent A*β*-induced astrocytes and microglia activation, and suppress inflammatory responses in the transgenic AD model [14]. *β*-sitosterol belongs to the group of phytosterols which are active ingredients existing in a diversity of natural plants. It is reported that *β*-sitosterol bound to the active sites of AChE and BChE as an inhibitor by molecular docking and exhibited an IC_50_ value of 55 and 50 *μ*g/ml against these two enzyme activities [28]. In addition, administration of *β*-sitosterol at 10 mg/kg body weight/day demonstrated gradual improvement in memory deficiency and motor coordination in the transgenic AD model [28]. Therefore, the above active components indicate the effectiveness and diversity of chemical ingredients in PLP for treating AD.

The results of PLP potential target-AD target network analysis acquired 33 key targets of PLP acting on AD. These key targets were mainly involved in biological processes such as regulation of transcription, regulation of apoptotic process, regulation of gene expression, inflammatory response, and regulation of nitric oxide biosynthetic process via molecular function of protein binding. The predicted results in our current study were consistent with some previous publications. It is reported that *β*-sitosterol attenuated A*β*-induced neural cell apoptosis and inflammation through downregulating iNOS expression and NF-*κ*B, p38, and ERK activation [29]. Kaempferol inhibited inflammatory response and iNOS expression as well as NO generation through suppressing NF-*κ*B, p38, JNK, and AKT phosphorylation [30]. Mairin promoted M2 phenotype microglial polarization and prevented M1 polarization through calmodulin-dependent protein kinase kinase *β*/AMPK activation, specifically decreased iNOS and TNF-*α* expression when the BV2 cells were treated with lipopolysaccharide (LPS) [31]. Further molecular docking assay in this study showed that *β*-sitosterol and mairin were conferred strong binding activity with MAPK1 (ERK2), MAPK8 (JNK1), NOS3, and TNF-*α*, while kaempferol had strong combination with AKT1 and TNF-*α*.

As observed in the results of KEGG pathway enrichment analysis, the key targets of PLP acting on AD were mainly related to PI3K-Akt signaling pathway, MAPK signaling pathway, neurotrophin signaling pathway, TNF signaling pathway, and so on. AD is an intricate neurodegenerative disease that the underlying mechanisms have not been clearly elucidated. It is recognized that the pathogenesis of AD is associated with various biological processes such as synaptic loss, specific neurotransmitters reduction, neuroinflammation, and neuronal death [32]. The neural cell death induced by aggregated A*β* plaque plays a crucial role in the pathogenesis of AD. The PI3K-Akt pathway participates in cell survival and death, particularly exhibiting beneficial effect on cell survival and inhibitory effect on cell apoptosis once Akt is activated [33]. In addition, PI3K-Akt pathway is involved in the initiation of the autophagic process which is a major intracellular machinery for degrading misfolded proteins and damaged organelles and has been reported to be involved in the pathogenesis of AD through acting on its downstream mTOR complex [34]. Notably, ubiquitin-proteasome system (UPS) is another major intracellular abnormal protein degradation system in eukaryotic cells that is likely associated with the etiology of AD. Ubiquitin possesses the function of labeling and binding to the proteins for degradation such as APP and *γ*-secretase activating protein that contributes to etiology of AD [35, 36]. It is found that aberrant form of this protein originating from misreading of UBB gene was accumulated in brain tissues of AD patients [37]. The molecular docking assay in this study showed that *β*-sitosterol, mairin, and palbinone presumably bound to UBB with strong affinity, implying the possible target of PLP on AD treatment.

MAPK pathway, the downstream signaling of multiple pathways, participated in synapse plasticity, neural cell survival, cell apoptosis, and neuroinflammation. A lot of studies have demonstrated that the crucial proteins of the MAPK signaling pathway such as ERK1, JNK, and p38 were all elevated in AD animal models, thus targeting these proteins could reduce A*β* production, tau phosphorylation, neuroinflammation, and synaptic loss, as well as slowed down the degeneration of cognitive function [38–40]. For example, Neurotropin® alleviated the accumulation of A*β* plaques and A*β*-induced neural cell death via suppression of HIF-1*α*, p-ERK1/2, p-JNK, and p-p38 in APP/PS1 mice [41]. Therefore, targeting MAPK signaling pathway-related proteins was a promising strategy for AD treatment.

BDNF and NGF are two of the pivotal factors involved in neurotrophin signaling pathway and play important roles in cholinergic synapse and synaptic plasticity. BDNF contributes to the development of hippocampal structure and function, while NGF promotes the function of cerebrum cholinergic neurons and prohibits neural cell death [42]. It is found that BDNF levels in peripheral blood and in cerebrospinal fluid as well as in hippocampus and neocortex of AD patients were significantly decreased compared with controls. However, NGF level in blood did not show evident change, but NGF levels in cerebrospinal fluid and hippocampus and neocortex of AD patients were significantly increased, suggesting that aberrations of neurotrophic factors were involved in the etiology and pathogenesis of AD [43]. Targeting neurotrophin signaling pathway to restore neural function was potential strategy for AD treatment [44]. The results of network analysis in this study also demonstrated that BDNF was one of the key targets related to the treatment of PLP for AD, and kaempferol had strong binding activity with BDNF by molecular docking.

In view of the pathological complexity of disease, herbal medicine can act on various molecules and targets to exert systematic actions on the disease. Network pharmacology is a powerful method to study the synergistic actions and underlying mechanisms of traditional medicines. However, it is a predicting method based on database analysis to find the possible mechanisms of drugs. Further biological studies are warranted to verify the above findings.

## 5. Conclusions

In conclusion, the present study discovered compound-target-disease interactions and possible mechanisms of PLP on AD treatment using network pharmacology strategy and predicted that *β*-sitosterol, kaempferol, lactiflorin, mairin, paeoniflorigenone, paeoniflorin, and palbinone were potential active ingredients of PLP, which possibly prevented AD via inhibiting neural cell apoptosis, inflammatory response, and promoting neurotrophy. It provided the theoretic elucidation of the ameliorative effect of PLP against AD and might facilitate the development of PLP or its active compounds as alternative therapy for AD.

## Figures and Tables

**Table 1:** The characteristics of active ingredients in PLP.

Compounds	Molecular formula	Structure	Molecular weight	OB (%)	HL	DL
*β*-sitosterol	C_29_H_50_O	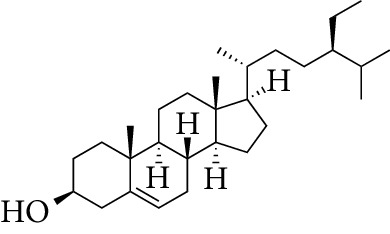	414.79	36.91	5.36	0.75

Kaempferol	C_15_H_10_O_6_	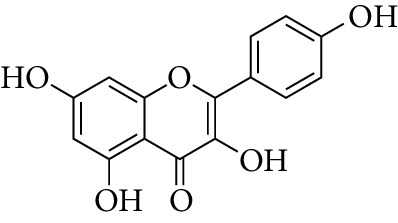	286.25	41.88	14.74	0.24

Lactiflorin	C_23_H_26_O_10_	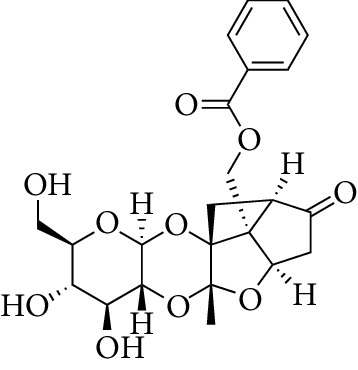	462.49	49.12	7.26	0.8

Mairin	C_30_H_48_O_3_	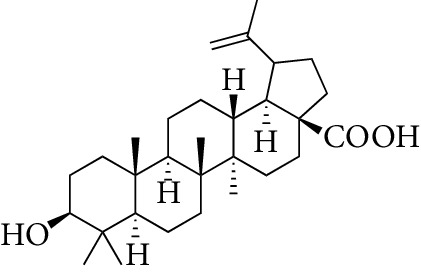	456.78	55.38	8.87	0.78

Paeoniflorigenone	C_17_H_18_O_6_	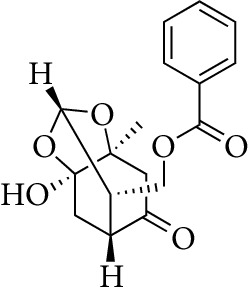	318.35	87.59	7.45	0.37

Paeoniflorin	C_23_H_28_O_11_	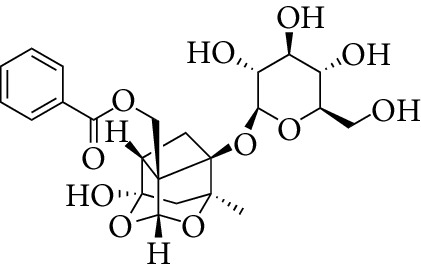	480.51	53.87	13.88	0.79

Palbinone	C_22_H_30_O_4_	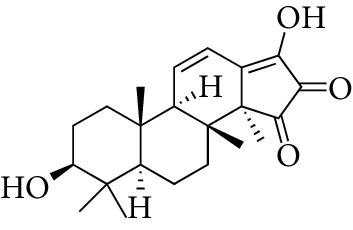	358.52	43.56	4.34	0.53

**Table 2:** The 33 key targets of PLP acting on AD.

Target	Name	Degree	Betweenness centrality	Closeness centrality
APP	Amyloid precursor protein	81	0.192	0.517
TP53	Tumor protein P53	54	0.096	0.507
AKT1	RAC-alpha serine/threonine-protein kinase	54	0.039	0.483
MAPK8	Mitogen-activated protein kinase 8	51	0.042	0.489
IL6	Interleukin 6	50	0.035	0.485
INS	Insulin	47	0.060	0.490
TNF	Tumor necrosis factor	46	0.022	0.473
MAPK1	Mitogen-activated protein kinase 1	45	0.036	0.481
JUN	Transcription factor AP1	44	0.026	0.482
VEGFA	Vascular endothelial growth factor A	43	0.029	0.475
APOE	Apolipoprotein E	38	0.040	0.458
IL8	Interleukin 8	38	0.028	0.444
BDNF	Brain-derived neurotrophic factor	37	0.039	0.465
CASP3	Caspase 3	35	0.023	0.461
IL1B	Interleukin-1*β*	35	0.016	0.443
RELA	Transcription factor p65	33	0.012	0.456
UBB	Ubiquitin B	32	0.032	0.441
HSP90A	Heat shock protein HSP 90	32	0.031	0.431
NOS3	Endothelial nitric oxide synthase	30	0.018	0.449
AGTR1	Type-1 angiotensin II receptor	29	0.016	0.437
PTGS2	Prostaglandin G/H synthase 2	29	0.026	0.435
NGF	Nerve growth factor	28	0.013	0.443
PPARG	Peroxisome proliferator activated receptor *γ*	28	0.013	0.434
AR	Androgen receptor	25	0.026	0.421
SP1	Sp1 transcription factor	24	0.014	0.424
CASP8	Caspase 8	22	0.007	0.436
PSEN1	Presenilin 1	22	0.015	0.427
CDK5	Cyclin-dependent kinase 5	22	0.013	0.424
NGFR	Nerve growth factor receptor	21	0.011	0.440
CAT	Catalase	21	0.020	0.423
MAPT	Microtubule associated protein tau	20	0.010	0.422
LDLR	Low-density lipoprotein receptor	20	0.020	0.417
GSK3B	Glycogen synthase kinase-3*β*	17	0.006	0.433

## Data Availability

The data used to support the findings of this study are available from the corresponding author upon request.

## Supplementary Materials

Supplementary Materials The characteristics of 85 compounds in PLP are shown in Supplementary Table 1. The predicted targets of *β*-sitosterol, kaempferol, lactiflorin, mairin, paeoniflorin, palbinone, and paeoniflorigenone are listed in Supplementary Table 2. The acknowledged targets of Alzheimer disease are displayed in Supplementary Table 3.
