# Supplementary material for: A Network Pharmacology Approach to Reveal the Underlying Mechanisms of Paeonia lactiflora Pall. On the Treatment of Alzheimer's Disease
Source: Evid Based Complement Alternat Med. 2019 Nov 16;2019:8706589. doi: 10.1155/2019/8706589 (PMC6885190; doi:10.1155/2019/8706589)
Supplement: Supplementary Materials — The characteristics of 85 compounds in PLP are shown in Supplementary Table 1. The predicted targets of β-sitosterol, kaempferol, lactiflorin, mairin, paeoniflorin, palbinone, and paeoniflorigenone are listed in Supplementary Table 2. The acknowledged targets of Alzheimer disease are displayed in Supplementary Table 3. [file 8706589.f1.pdf]

Supplementary materials

Stable 1. The characteristics of compounds in PLP

Name	Mol ID	MW	OB%	DL	HL	BBB
MOL001934	(3R,3aR,6S,7aR)-6-hydroxy-3,6-dimethyl-3a,4,7,7a-tetrahydro-3H-benzofuran-2,5-dione	198.24	104.94	0.08	13.69	-0.32
MOL001935	(3aR,6S,7aR)-6-hydroxy-6-methyl-3-methylene-3a,4,7,7a-tetrahydrobenzofuran-2,5-dione	196.22	97.79	0.08	13.98	-0.13
MOL001917	(3S,3aR,5S,6S,7aR)-5,6-dihydroxy-3,6-dimethyl-3,3a,4,5,7,7a-hexahydrobenzofuran-2-one	200.26	96.64	0.07	13.6	-0.69
MOL001918	paeoniflorgenone	318.35	87.59	0.37	7.45	-0.56
MOL001931	4-Chlorobutyric acid	122.56	85.82	0.01	11.6	0.85
MOL008707	Ethylisobutyrate	116.18	83.67	0.01	11.14	1.49
MOL001888	2,2-dimethylcyclohexanol	128.24	82.54	0.02	11.06	1.6
MOL001937	24253-30-3	98.16	74.2	0.01	11.36	1.57
MOL001925	paeoniflorin_qt	318.35	68.18	0.4	8.81	-0.73
MOL001928	albiflorin_qt	318.35	66.64	0.33	6.54	-0.88
MOL001892	Dipropyl phthalate	250.32	66.3	0.1	5.7	0.71
MOL001910	11α,12α-epoxy-3β-23-dihydroxy-30-norolean-20-en-28,12β-olide	470.71	64.77	0.38	2.62	-0.59
MOL000676	DBP	278.38	64.54	0.13	5.41	0.56
MOL001246	(1R)-()-Nopinone	138.23	57.86	0.05	11.1	1.6
MOL000612	(-)-α-cedrene	204.39	55.56	0.1	4.82	2.16
MOL000211	Mairin	456.78	55.38	0.78	8.87	0.22
MOL000492	(+)-catechin	290.29	54.83	0.24	0.61	-0.73
MOL001924	paeoniflorin	480.51	53.87	0.79	13.88	-1.86
MOL001904	2 - methyl - 3 - (2 - propenyl) - phenol	148.22	52.06	0.03	4.7	1.81
MOL001909	(Z)-(1S,5R)-β-pinen-10-yl-β-vicianoside_qt	152.26	50.32	0.06	11.47	1.93
MOL001896	bicyclo[3.1.1]hept-2-ene-2-methanol, 6,6-dimethyl-	152.26	49.79	0.06	11.54	1.34
MOL001897	()-trans-Myrtanol	154.28	49.66	0.06	11.13	1.37
MOL001921	Lactiflorin	462.49	49.12	0.8	7.26	-1.76
MOL004480	acetic acid	60.06	47.87	0	12.21	0.72
MOL001898	Acetyl oxide	102.1	45.13	0.01	11.84	0.77
MOL000775	EEE	88.12	45.02	0	11.64	1.26
MOL001919	(3S,5R,8R,9R,10S,14S)-3,17-dihydroxy-4,4,8,10,14-pentamethyl-2,3,5,6,7,9-hexahydro-1H-cyclopenta[a]phenanthrene-15,16-dione	358.52	43.56	0.53	4.34	-0.41

MOL001889	Methyl linolelaidate	294.53	41.93	0.17	5.78	1.11
MOL000422	kaempferol	286.25	41.88	0.24	14.74	-0.55
MOL001895	Dibutylphenol	206.36	38.9	0.06	10.76	1.87
MOL000358	β-sitosterol	414.79	36.91	0.75	5.36	0.99
MOL000359	sitosterol	414.79	36.91	0.75	5.37	0.87
MOL001893	BU3	90.14	34.87	0.01	11.25	-0.01
MOL001801	salicylic acid	138.13	32.13	0.03	12	0.63
MOL001936	cis-5-Octen-1-ol	128.24	31.84	0.01	5.4	1.15
MOL000513	3,4,5-trihydroxybenzoic acid	170.13	31.69	0.04	11.78	-0.54
MOL000219	BOX	121.12	31.55	0.02	12.07	0.84
MOL001930	benzoyl paeoniflorin	584.62	31.27	0.75	-1.85	-1.24
MOL001906	Methylgallate	184.16	30.91	0.05	5.67	0.06
MOL000263	oleanolic acid	456.78	29.02	0.76		0.07
MOL000874	paeonol	166.19	28.79	0.04		0.84
MOL001219	Satol	268.54	27.27	0.11		0.97
MOL001891	9-methylenefluorene	178.24	26.87	0.09		1.78
MOL001912	albiflorin R1_qt	318.35	26.18	0.34		-0.85
MOL001907	Progallin A	198.19	25.61	0.06		0.11
MOL001913	propyl (2R)-2-hydroxypropanoate	132.18	25.5	0.01		0.35
MOL000876	(6R,10R)-6,10,14-trimethylpentadecan-2-one	268.54	23.3	0.1		1.35
MOL001899	ZINC02169908	268.54	23.3	0.1		1.24
MOL000106	PYG	126.12	22.98	0.02		0.79
MOL000551	Hederagenol	472.78	22.42	0.74		-0.51
MOL001933	oxypaeoniflorin	496.51	21.88	0.78		-2.41
MOL001902	3β,23-dihydroxy-oleana-11,13(18)-dien-28-oic acid	470.76	21.53	0.75		-0.62
MOL001644	Dodecanal	184.36	21.52	0.03		1.42
MOL001911	albiflorin R1	480.51	21.29	0.82		-1.98
MOL001393	myristic acid	228.42	21.18	0.07		0.99
MOL001929	alexandrin	576.95	20.63	0.62		-0.98
MOL000357	Sitogluside	576.95	20.63	0.62		-0.93
MOL001396	PENTADECYLIC ACID	242.45	20.18	0.08		0.88
MOL001890	octadec-9-ene	252.54	19.5	0.09		1.84
MOL001218	Pisol	186.38	18.5	0.03		1.06
MOL000610	TRD	184.41	17.89	0.03		1.96
MOL001923	[(3S,3aR,6S,7aR)-6-hydroxy-6-methyl-2,5-dioxo-3a,4,7,7a-tetrahydro-3H-benzofuran-3-yl]methyl benzoate	318.35	17.84	0.3		-0.66
MOL000860	stearic acid	284.54	17.83	0.14		1.22
MOL001905	3β-hydroxy-oleana-11,13(18)-dien-28-oic acid?	454.76	17.11	0.76		-0.13
MOL001922	2-Hexyl-1-decanol	242.5	17.08	0.07		1.03
MOL001900	Pulchinenoside A_qt	472.78	16.91	0.77		-0.58

MOL000875	Cedrol	222.41	16.23	0.12	1.46
MOL000561	Astragalin	448.41	14.03	0.74	-1.97
MOL001903	3β-hydroxy-11-oxo-olean-12-en-28-oic acid	470.76	13.49	0.74	-0.2
MOL001927	albiflorin	480.51	12.09	0.77	-2.19
MOL001901	24-Methylenecycloartanol	440.83	10.4	0.79	1.13
MOL007465	10-Methylnonadecane	282.62	10.28	0.12	1.79
MOL000867	Heptadekan	240.53	8.64	0.07	1.87
MOL000868	LFA	282.62	8.46	0.13	1.8
MOL000869	Henicosane	296.65	8.41	0.15	1.8
MOL005089	Oxypaeoniflorin	496.51	8.38	0.78	-2.33
MOL001402	Octacosane	394.86	8.15	0.37	1.46
MOL001894	Bicetyl	450.98	8.03	0.46	1.71
MOL001914	gallotannin	1701.27	7.36	0.03	-7.15
MOL000842	sucrose	342.34	7.17	0.23	-6.67
MOL001908	(Z)-(1S,5R)-β-pinen-10-yl-β-vicianoside	446.55	5.74	0.67	-1.89
MOL001915	paeonoside	610.57	3.47	0.71	-3.73
MOL001932	galloylpaeoniflorin	632.62	3.03	0.42	-2.78
MOL001926	1,2,3,6-tetra-O-galloylglucose	788.62	3.01	0.34	-4.03
MOL009092	Pentagalloylglucose	940.72	3.01	0.21	-4.17

Stable 2. The compound-related targets

**Compounds**	**Targets**	**Uniprot ID**	**Gene name**
β-sitosterol	Tyrosyl-DNA phosphodiesterase 1	Q9NUW8	TDP1
β-sitosterol	Androgen receptor	P10275	AR
β-sitosterol	3-hydroxy-3-methylglutaryl-coenzyme A reductase	P04035	HMGCR
β-sitosterol	Lanosterol 14-α demethylase	Q16850	CYP51A1
β-sitosterol	Oxysterols receptor LXR-β	P55055	NR1H2
β-sitosterol	Oxysterols receptor LXR-α	Q13133	NR1H3
β-sitosterol	Cytochrome P450 19A1	P11511	CYP19A1
β-sitosterol	Steroid 17-α-hydroxylase/17	P05093	CYP17A1
β-sitosterol	Low-density lipoprotein receptor	P01130	LDLR
β-sitosterol	Very low-density lipoprotein receptor	P98155	VLDLR
β-sitosterol	Low-density lipoprotein receptor-related protein 8	Q14114	LRP8
β-sitosterol	Estrogen receptor	P03372	ESR1
β-sitosterol	Estrogen receptor β	Q92731	ESR2
β-sitosterol	Sodium-dependent noradrenaline transporter	P23975	SLC6A2
β-sitosterol	Sodium-dependent serotonin transporter	P31645	SLC6A4
β-sitosterol	Nuclear receptor coactivator 2	Q15596	NCOA2

β-sitosterol	Prostaglandin G/H synthase 1	P23219	PTGS1
β-sitosterol	Prostaglandin G/H synthase 2	P35354	PTGS2
β-sitosterol	Heat shock protein HSP 90	P07900	HSP90
β-sitosterol	Phosphatidylinositol-4,5-bisphosphate 3-kinase catalytic subunit, γ isoform	P48736	PIK3CG
β-sitosterol	mRNA of PKA Catalytic Subunit C-α	P17612	PRKACA
β-sitosterol	Muscarinic acetylcholine receptor M1	P11229	CHRM1
β-sitosterol	γ-aminobutyric-acid receptor α-2 subunit	P47869	GABRA2
β-sitosterol	Muscarinic acetylcholine receptor M2	P08172	CHRM2
β-sitosterol	A-1B adrenergic receptor	P35368	ADRA1B
β-sitosterol	Apoptosis regulator Bcl-2	P10415	BCL2
β-sitosterol	Apoptosis regulator BAX	Q07812	BAX
β-sitosterol	Transcription factor AP-1	P05412	JUN
β-sitosterol	Potassium voltage-gated channel subfamily H member 2	Q12809	KCNH2
β-sitosterol	Dopamine D1 receptor	P21728	DRD1
β-sitosterol	Muscarinic acetylcholine receptor M3	P20309	CHRM3
β-sitosterol	Sodium channel protein type 5 subunit α	Q14524	SCN5A
β-sitosterol	Muscarinic acetylcholine receptor M4	P08173	CHRM4
β-sitosterol	CGMP-inhibited 3',5'-cyclic phosphodiesterase A	Q14432	PDE3A
β-sitosterol	5-hydroxytryptamine 2A receptor	P28223	HTR2A
β-sitosterol	γ-aminobutyric-acid receptor α-5 subunit	P31644	GABRA5
β-sitosterol	A-1A adrenergic receptor	P35348	ADRA1A
β-sitosterol	γ-aminobutyric-acid receptor α-3 subunit	P34903	GABRA3
β-sitosterol	B-2 adrenergic receptor	P07550	ADRB2
β-sitosterol	Neuronal acetylcholine receptor subunit α-2	Q15822	CHRNA2
β-sitosterol	Mu-type opioid receptor	P35372	OPRM1
β-sitosterol	Neuronal acetylcholine receptor protein, α-7 chain	P36544	CHRNA7
β-sitosterol	Caspase-9	P55211	CASP9
β-sitosterol	Caspase-8	Q14790	CASP8
β-sitosterol	Transforming growth factor β-1	P01137	TGFB1
β-sitosterol	Serum paraoxonase/arylesterase 1	P27169	PON1
β-sitosterol	Microtubule-associated protein 2	P11137	MAP2
β-sitosterol	Sterol regulatory element binding transcription factor 2	Q12772	SREBF2
β-sitosterol	ATP-binding cassette, sub-family G (WHITE), member 8	Q9H221	ABCG8
β-sitosterol	ATP-binding cassette, sub-family G (WHITE), member 5	Q9H222	ABCG5
β-sitosterol	Apolipoprotein E	P02649	APOE
β-sitosterol	24-dehydrocholesterol reductase	Q15392	DHCR24
β-sitosterol	Caspase 3	P42574	CASP3
β-sitosterol	Sterol regulatory element binding transcription	P36956	SREBF1

	factor 1		
β-sitosterol	ATP-binding cassette, sub-family B (MDR/TAP), member 11	O95342	ABCB11
β-sitosterol	Intercellular adhesion molecule 1	P05362	ICAM1
β-sitosterol	Cytochrome P450, family 7, subfamily A, polypeptide 1	P22680	CYP7A1
Kaempferol	Cytochrome P450 1A2	P05177	CYP1A2
Kaempferol	Multidrug resistance protein 1	P08183	ABCB1
Kaempferol	Arachidonate 5-lipoxygenase	P09917	ALOX5
Kaempferol	Estradiol 17-β-dehydrogenase 1	P14061	HSD17B1
Kaempferol	Aldose reductase	P15121	AKR1B1
Kaempferol	Arachidonate 15-lipoxygenase	P16050	ALOX15
Kaempferol	Arachidonate 12-lipoxygenase	P18054	ALOX12
Kaempferol	Multidrug resistance-associated protein 1	P33527	ABCC1
Kaempferol	Estradiol 17-β-dehydrogenase 2	P37059	HSD17B2
Kaempferol	Xanthine dehydrogenase/oxidase	P47989	XDH
Kaempferol	Dual specificity tyrosine-phosphorylation-regulated kinase 1A	Q13627	DYRK1A
Kaempferol	NADPH oxidase 4	Q9NPH5	NOX4
Kaempferol	Tyrosyl-DNA phosphodiesterase 1	Q9NUW8	TDP1
Kaempferol	Nitric oxide synthase, inducible	P35228	NOS2
Kaempferol	Peroxisome proliferator activated receptor γ	P37231	PPARG
Kaempferol	Dipeptidyl peptidase IV	P27487	DPP4
Kaempferol	Trypsin-1	P07477	PRSS1
Kaempferol	Thrombin	P00734	F2
Kaempferol	Nitric oxide synthase, endothelial	P29474	NOS3
Kaempferol	Acetylcholinesterase	P22303	ACHE
Kaempferol	DNA topoisomerase II	P11388	TOP2
Kaempferol	Coagulation factor VII	P08709	F7
Kaempferol	Calmodulin	P0DP23	CALM
Kaempferol	Transcription factor p65	Q04206	RELA
Kaempferol	Inhibitor of nuclear factor kappa-B kinase subunit β	O14920	IKBKB
Kaempferol	RAC-α serine/threonine-protein kinase	P31749	AKT1
Kaempferol	Activator of 90 kDa heat shock protein ATPase homolog 1	O95433	AHSA1
Kaempferol	Mitogen-activated protein kinase 8	P45983	MAPK8
Kaempferol	Interstitial collagenase	P03956	MMP1
Kaempferol	Signal transducer and activator of transcription 1-α/β	P42224	STAT1
Kaempferol	Heme oxygenase 1	P09601	HMOX1
Kaempferol	Cytochrome P450 3A4	P08684	CYP3A4
Kaempferol	Cytochrome P450 1A1	P04798	CYP1A1
Kaempferol	Intercellular adhesion molecule 1	P05362	ICAM1

Kaempferol	E-selectin	P16581	SELE
Kaempferol	Vascular cell adhesion protein 1	P19320	VCAM1
Kaempferol	Hyaluronan synthase 2	Q92819	HAS2
Kaempferol	Glutathione S-transferase P	P09211	GSTP1
Kaempferol	26S proteasome non-ATPase regulatory subunit 3	O43242	PSMD3
Kaempferol	Solute carrier family 2, facilitated glucose transporter member 4	P14672	GLUT4
Kaempferol	Nuclear receptor subfamily 1 group I member 3	Q14994	NR1I3
Kaempferol	Insulin receptor	P06213	INSR
Kaempferol	Type I iodothyronine deiodinase	P49895	DIO1
Kaempferol	Serine/threonine-protein phosphatase 2B catalytic subunit α isoform	Q08209	PPP3CA
Kaempferol	Peroxidase C1A	P00433	PRXC1A
Kaempferol	Glutathione S-transferase Mu 1	P09488	GSTM1
Kaempferol	Glutathione S-transferase Mu 2	P28161	GSTM2
Kaempferol	Aldo-keto reductase family 1 member C3	P42330	AKR1C3
Kaempferol	Antileukoproteinase	P03973	SLPI
Kaempferol	Cyclin-dependent kinase 1	P06493	CDK1
Kaempferol	Nuclear receptor subfamily 1, group I, member 2	O75469	NR1I2
Kaempferol	Cytochrome P450, family 1, subfamily B, polypeptide 1	Q16678	CYP1B1
Kaempferol	Ribosomal protein S6 kinase α-3	P51812	RPS6KA3
Kaempferol	Aryl hydrocarbon receptor	P35869	AHR
Kaempferol	UDP glucuronosyltransferase 1 family, polypeptide A3	P35503	UGT1A3
Kaempferol	UDP glucuronosyltransferase 1 family, polypeptide A8	Q9HAW9	UGT1A8
Kaempferol	UDP glucuronosyltransferase 1 family, polypeptide A7	Q9HAW7	UGT1A7
Kaempferol	UDP glucuronosyltransferase 1 family, polypeptide A9	O60656	UGT1A9
Lactiflorin	Galectin-9	O00182	LGALS9
Lactiflorin	Galectin-3	P17931	LGALS3
Lactiflorin	Galectin-9B	Q3B8N2	LGALS9B
Lactiflorin	Galectin-9C	Q6DKI2	LGALS9C
Lactiflorin	Platelet-activating factor receptor	P25105	PTAFR
Mairin	Aldo-keto reductase family 1 member B10	O60218	AKR1B10
Mairin	DNA polymerase β	P06746	POLB
Mairin	Aldose reductase	P15121	AKR1B1
Mairin	Aldo-keto reductase family 1 member B15	C9JRZ8	AKR1B15
Mairin	Alcohol dehydrogenase [NADP(+)]	P14550	AKR1A1
Mairin	5-anhydro-D-fructose reductase	Q96JD6	AKR1E2
Mairin	Microtubule-associated protein tau	P10636	MAPT
Mairin	Corticosteroid 11-β-dehydrogenase isozyme 1	P28845	HSD11B1

Mairin	Hydroxysteroid 11-β-dehydrogenase 1-like protein	Q7Z5J1	HSD11B1L
Mairin	Bile acid receptor	Q96RI1	NR1H4
Mairin	M-phase inducer phosphatase 1	P30304	CDC25A
Mairin	M-phase inducer phosphatase 2	P30305	CDC25B
Mairin	DNA topoisomerase 2-α	P11388	TOP2A
Mairin	DNA topoisomerase 2-β	Q02880	TOP2B
Mairin	Progesterone receptor	P06401	PGR
Mairin	Cytochrome c, somatic	P99999	CYCS
Mairin	Pancreatic lipase	P16233	PNLIP
Mairin	Sp1 transcription factor	P08047	SP1
Mairin	Nitric oxide synthase 3，*endothelial*	P29474	NOS3
Mairin	Lamin B1	P20700	LMNB1
Mairin	Caspase 3	P42574	CASP3
Mairin	v-akt murine thymoma viral oncogene homolog 1	P31749	AKT1
Mairin	Baculoviral IAP repeat containing 5	O15392	BIRC5
Mairin	Topoisomerase (DNA) I	P11387	TOP1
Paeoniflorigenone	γ-aminobutyric acid receptor subunit α-1	P14867	GABRA1
Paeoniflorin	Galectin-9	O00182	LGALS9
Paeoniflorin	Galectin-3	P17931	LGALS3
Paeoniflorin	Galectin-9B	Q3B8N2	LGALS9B
Paeoniflorin	Galectin-9C	Q6DKI2	LGALS9C
Paeoniflorin	Sodium-dependent noradrenaline transporter	P23975	SLC6A2
Paeoniflorin	Sodium- and chloride-dependent glycine transporter 1	P48067	SLC6A9
Paeoniflorin	Sodium-dependent dopamine transporter	Q01959	SLC6A3
Paeoniflorin	Sodium-dependent serotonin transporter	P31645	SLC6A4
Paeoniflorin	Sodium-dependent proline transporter	Q99884	SLC6A7
Paeoniflorin	Sodium- and chloride-dependent neutral and basic amino acid transporter B(0+)	Q9UN76	SLC6A14
Paeoniflorin	Sodium- and chloride-dependent glycine transporter 2	Q9Y345	SLC6A5
Paeoniflorin	Interleukin-6	P05231	IL6
Paeoniflorin	Monocyte differentiation antigen CD14	P08571	CD14
Paeoniflorin	Tumor necrosis factor	P01375	TNF
Paeoniflorin	Lipopolysaccharide-binding protein	P18428	LBP
Paeoniflorin	Heat shock transcription factor 1	Q00613	HSF1
Paeoniflorin	Toll-like receptor 4	O00206	TLR4
Paeoniflorin	Interleukin 8	P10145	IL8
Palbinone	Androgen receptor	P10275	AR
Palbinone	Progesterone receptor	P06401	PGR
Palbinone	Mineralocorticoid receptor	P08235	NR3C2

Stable 3. The AD-related targets

Gene name	Target name	UniProt ID
HTR1A	5-hydroxytryptamine 1A receptor	P08908
HTR4	5-hydroxytryptamine 4 receptor	Q13639
ADRA2C	A-2C adrenergic receptor	P18825
MAOA	Amine oxidase [flavin-containing] A	P21397
MAOB	Amine oxidase [flavin-containing] B	P27338
GABRA1	γ-aminobutyric acid receptor subunit α-1	P14867
GABRA2	γ-aminobutyric acid receptor subunit α-2	P47869
GABRB2	γ-aminobutyric acid receptor subunit β-2	P47870
HPGDS	Glutathione S-transferase	O60760
INSR	Insulin receptor	P06213
CHRM4	Muscarinic acetylcholine receptor M5	P08173
CHRM3	Muscarinic acetylcholine receptor M3	P20309
CHRM1	Muscarinic acetylcholine receptor M1	P11229
CHRM2	Muscarinic acetylcholine receptor M2	P08172
CHRNB2	Nicotinic ACh receptor β 2	P17787
CHRNA4	Nicotinic ACh receptor α 4	P43681
GRIN1	NMDA receptor	Q05586
TSPO	Peripheral-type benzodiazepine receptor	P30536
PTGS1	Prostaglandin G/H synthase 1	P23219
SSTR1	Somatostatin receptor 1	P30872
SSTR2	Somatostatin receptor type 2	P30874
SSTR3	Somatostatin receptor type 3	P32745
SSTR5	Somatostatin receptor type 5	P35346
SLC18A2	Synaptic vesicle amine transporter	Q05940
AGTR1	Type-1 angiotensin II receptor	P30556
BACE1	β-secretase 1	P56817
BACE2	β-secretase 2	Q9Y5Z0
PSENEN	γ-secretase	Q9NZ42
CCR2	C-C chemokine receptor type 2	P41597
GRIA1	Glutamate receptor 1	P42261
HRH3	Histamine H3 receptor	Q9Y5N1
GRM1	Metabotropicglutamate receptor 1	Q13255
GRM2	Metabotropic glutamate receptor 2	Q14416
GRM5	Metabotropic glutamate receptor 5	P41594
PTGES	Prostaglandin E synthase	O14684
NGFR	Tumor necrosis factor receptor superfamily member 16	P08138
SNCA	A-synuclein	P37840
ACHE	Acetylcholinesterase	P22303
APP	Amyloid β A4 protein	P05067
DRD2	D(2) dopamine receptor	P14416
CHRNA7	Neuronal acetylcholine receptor protein, α-7 chain	P36544

PTGS2	Prostaglandin G/H synthase 2	P35354
GSK3B	Glycogen synthase kinase-3 β	P49841
MICAL2	Microtubule associated monooxygenase, calponin and LIM domain containing 2	O94851
DYNC1I1	Dynein, cytoplasmic 1, intermediate chain 1	O14576
RPH3A	Rabphilin 3A	Q9Y2J0
ATP2B1	ATPase, Ca2+ transporting, plasma membrane 1	P20020
ELMOD1	ELMO/CED-12 domain containing 1	Q8N336
NELL2	Protein kinase C-binding protein NELL2	Q99435
RASGRF1	Ras protein-specific guanine nucleotide-releasing factor 1	Q13972
CAMKK2	Calcium/calmodulin-dependent protein kinase kinase 2	Q96RR4
ICAM5	Intercellular adhesion molecule 5	Q9UMF0
PAK1	p21 protein (Cdc42/Rac)-activated kinase 1	Q13153
PSEN1	Presenilin 1	P49768
APOE	Apolipoprotein E	P02649
PSEN2	Presenilin 2	P49810
MAPT	Microtubule Associated Protein Tau	P10636
TNF	Tumor Necrosis Factor	P01375
PRNP	Prion Protein	P04156
IL1B	Interleukin 1β	P01584
ACE	Angiotensin I Converting Enzyme	P12821
NOS3	Nitric Oxide Synthase 3	P29474
VEGFA	Vascular Endothelial Growth Factor A	P15692
BDNF	Brain Derived Neurotrophic Factor	P23560
SORL1	Sortilin Related Receptor 1	Q92673
ABCA1	ATP Binding Cassette Subfamily A Member 1	O95477
SQSTM1	Sequestosome 1	Q13501
CTSD	Cathepsin D	P07339
GFAP	Glial Fibrillary Acidic Protein	P14136
BCHE	Butyrylcholinesterase	P06276
SOD1	Superoxide Dismutase 1	P00441
TF	Transferrin	P02787
CHAT	Choline O-Acetyltransferase	P28329
ADAM10	ADAM Metallopeptidase Domain 10	O14672
HFE	Homeostatic Iron Regulator	Q30201
MPO	Myeloperoxidase	P05164
GDNF	Glial Cell Derived Neurotrophic Factor	P39905
SLC6A4	Solute Carrier Family 6 Member 4	P31645
FAS	Fas Cell Surface Death Receptor	P25445
GBA	Glucosylceramidase B	P04062
MME	Membrane Metalloendopeptidase	P08473
MTND1	Mitochondrially Encoded NADH:Ubiquinone Oxidoreductase Core Subunit 1	P03886
UCHL1	Ubiquitin C-Terminal Hydrolase L1	P09936

PPARG	Peroxisome Proliferator Activated Receptor Gamma	P37231
LRRK2	Leucine Rich Repeat Kinase 2	Q5S007
PLAU	Urokinase-type plasminogen activator	P00749
LRP1	LDL Receptor Related Protein 1	Q07954
PRKN	Parkin RBR E3 Ubiquitin Protein Ligase	O60260
CST3	Cystatin C	P01034
A2M	A-2-Macroglobulin	P01023
CAT	Catalase	P04040
MTHFR	Methylenetetrahydrofolate Reductase	P42898
TTR	Transthyretin	P02766
TARDBP	TAR DNA Binding Protein	Q13148
LPL	Lipoprotein Lipase	P06858
NGF	Nerve Growth Factor	P01138
IL6	Interleukin 6	P05231
INS	Insulin	P01308
MAPK1	Mitogen-Activated Protein Kinase 1	P28482
ABCA7	ATP Binding Cassette Subfamily A Member 7	Q8IZY2
HSD17B10	Hydroxysteroid 17-B Dehydrogenase 10	Q99714
LDLR	Low Density Lipoprotein Receptor	P01130
SYP	Synaptophysin	P08247
GRN	Granulin Precursor	P28799
HMOX1	Heme Oxygenase 1	P09601
TNFRSF1A	TNF Receptor Superfamily Member 1A	P19438
ADAM17	ADAM Metallopeptidase Domain 17	P78536
CD40	CD40 Molecule	P25942
MTND2	Mitochondrially Encoded NADH:Ubiquinone Oxidoreductase Core Subunit 2	P03891
SNCB	Synuclein B	Q16143
CASP8	Caspase 8	Q14790
CRH	Corticotropin Releasing Hormone	P06850
NCSTN	Nicastrin	Q92542
GRIN2B	Glutamate Ionotropic Receptor NMDA Type Subunit 2B	Q13224
PLD3	Phospholipase D Family Member 3	Q8IV08
NTRK2	Neurotrophic Receptor Tyrosine Kinase 2	Q16620
MTCO1	Mitochondrially Encoded Cytochrome C Oxidase I	P00395
NTRK1	Neurotrophic Receptor Tyrosine Kinase 1	P04629
RELN	Reelin	P78509
PARK7	Parkinsonism Associated Deglycase	Q99497
IL10	Interleukin 10	P22301
SLC6A3	Solute Carrier Family 6 Member 3	Q01959
OLR1	Oxidized Low Density Lipoprotein Receptor 1	P78380
SERPINA3	Serpin Family A Member 3	P01011
DKK1	Dickkopf WNT Signaling Pathway Inhibitor 1	O94907
CTSB	Cathepsin B	P07858

PICALM	Phosphatidylinositol Binding Clathrin Assembly Protein	Q13492
VLDLR	Very Low Density Lipoprotein Receptor	P98155
COL25A1	Collagen Type XXV A 1 Chain	Q9BXS0
NPC1	NPC Intracellular Cholesterol Transporter 1	O15118
UBB	Ubiquitin B	P0CG47
DNM1L	Dynamin 1 Like protein	O00429
CDK5	Cyclin Dependent Kinase 5	Q00535
CLU	Clusterin	P10909
GRIN2A	Glutamate Ionotropic Receptor NMDA Type Subunit 2A	Q12879
IL1A	Interleukin 1α	P01583
COX5A	Cytochrome C Oxidase Subunit 5A	P20674
DHCR24	24-Dehydrocholesterol Reductase	Q15392
CAPN1	Calpain 1	P07384
CASP3	Caspase 3	P42574
COMT	Catechol-O-Methyltransferase	P21964
ITM2B	Integral Membrane Protein 2B	Q9Y287
MTCO2	Mitochondrially Encoded Cytochrome C Oxidase II	P00403
DYRK1A	Dual Specificity Tyrosine Phosphorylation Regulated Kinase 1A	Q13627
ITPR1	Inositol 1,4,5-Trisphosphate Receptor Type 1	Q14643
MSR1	Macrophage Scavenger Receptor 1	P21757
SEMA3A	Semaphorin 3A	Q14563
RYR1	Ryanodine Receptor 1	P21817
CALM	Calmodulin 1	P0DP23
AKT1	AKT Serine/Threonine Kinase 1	P31749
NOD2	Nucleotide Binding Oligomerization Domain Containing 2	Q9HC29
TGFB1	Transforming Growth Factor β1	P01137
HTT	Huntingtin	P42858
TREM2	Triggering Receptor Expressed On Myeloid Cells 2	Q9NZC2
EIF2AK3	Eukaryotic Translation Initiation Factor 2 A Kinase 3	Q9NZJ5
IFNG	Interferon γ	P01579
SLC1A3	Solute Carrier Family 1 Member 3	P43003
TP53	Tumor Protein P53	P04637
HTR2A	5-Hydroxytryptamine Receptor 2A	P28223
ATP7B	ATPase Copper Transporting B	P35670
